# RETRACTION: Alpha B‐Crystallin Promotes the Invasion and Metastasis of Gastric Cancer via NF‐κB‐Induced Epithelial‐Mesenchymal Transition

**DOI:** 10.1111/jcmm.70369

**Published:** 2025-01-13

## Abstract

RETRACTION: D. Chen, G. Cao, C. Qiao, G. Liu, H. Zhou, and Q. Liu, “Alpha B‐Crystallin Promotes the Invasion and Metastasis of Gastric Cancer via NF‐κB‐Induced Epithelial‐Mesenchymal Transition,” *Journal of Cellular and Molecular Medicine* 22, no. 6 (2018): 3215–3222, https://doi.org/10.1111/jcmm.13602.

The above article, published online on 22 March 2018 in Wiley Online Library (wileyonlinelibrary.com), has been retracted by agreement between the journal Editor‐in‐Chief, Stefan N. Constantinescu; the Foundation for Cellular and Molecular Medicine; and John Wiley & Sons Ltd. The retraction has been agreed due to concerns raised by third parties. The article presents experiments in an unverifiable/unknown cell line, MGC823. Furthermore, additional flaws and inconsistencies between results presented and experimental methods described were found. Accordingly, the article is retracted as its conclusions are considered invalid by the editors. The authors did not respond to our requests for clarification regarding the identified issues and have been informed of the decision of retraction.

RETRACTION: D. Chen, G. Cao, C. Qiao, G. Liu, H. Zhou, and Q. Liu, “Alpha B‐Crystallin Promotes the Invasion and Metastasis of Gastric Cancer via NF‐κB‐Induced Epithelial‐Mesenchymal Transition,” *Journal of Cellular and Molecular Medicine* 22, no. 6 (2018): 3215–3222, https://doi.org/10.1111/jcmm.13602.

The above article, published online on 22 March 2018 in Wiley Online Library (wileyonlinelibrary.com), has been retracted by agreement between the journal Editor‐in‐Chief, Stefan N. Constantinescu; the Foundation for Cellular and Molecular Medicine; and John Wiley & Sons Ltd. The retraction has been agreed due to concerns raised by third parties. The article presents experiments in an unverifiable/unknown cell line, MGC823. Furthermore, additional flaws and inconsistencies between results presented and experimental methods described were found. Accordingly, the article is retracted as its conclusions are considered invalid by the editors. The authors did not respond to our requests for clarification regarding the identified issues and have been informed of the decision of retraction.

